# The SGLT2 Inhibitor Empagliflozin Ameliorates the Inflammatory Profile in Type 2 Diabetic Patients and Promotes an Antioxidant Response in Leukocytes

**DOI:** 10.3390/jcm8111814

**Published:** 2019-11-01

**Authors:** Francesca Iannantuoni, Aranzazu M. de Marañon, Noelia Diaz-Morales, Rosa Falcon, Celia Bañuls, Zaida Abad-Jimenez, Victor M. Victor, Antonio Hernandez-Mijares, Susana Rovira-Llopis

**Affiliations:** 1 Service of Endocrinology and Nutrition, University Hospital Doctor Peset, Foundation for the Promotion of Health and Biomedical Research in the Valencian Region (FISABIO), 46017 Valencia, Spain; franian@alumni.uv.es (F.I.); arantxa.mmp@gmail.com (A.M.d.M.); noeliadm_89@hotmail.com (N.D.-M.); rosafalcon16@gmail.com (R.F.); celia.banuls@gmail.com (C.B.); zaiaji@alumni.uv.es (Z.A.-J.); 2 CIBERehd-Department of Pharmacology and Physiology, University of Valencia, 46010 Valencia, Spain; 3 Department of Physiology, University of Valencia, 46010 Valencia, Spain; 4 Institute of Health Research INCLIVA, University of Valencia, 46010 Valencia, Spain; 5 Department of Medicine, University of Valencia, 46010 Valencia, Spain

**Keywords:** empagliflozin, SGLT2 inhibitors, type 2 diabetes, oxidative stress, inflammation, leukocytes

## Abstract

Sodium–glucose co-transporter 2 inhibitors (iSGLT2) have been linked to a considerable reduction in cardiovascular risk in patients with type 2 diabetes (T2D), but the precise molecular mechanisms are still elusive. We aimed to evaluate the effects of the iSGLT2 empagliflozin on systemic inflammation and its potential antioxidant properties. This is an observational, prospective follow-up study of a cohort of fifteen patients with T2D who received 10 mg/day of empagliflozin according to standard clinical care. Measures at baseline, 12 and 24 weeks were taken. Metabolic and anthropometric parameters were evaluated. Production of mitochondrial superoxide, glutathione content, and glutathione s-reductase and catalase mRNA levels were measured in leukocytes. Serum levels of myeloperoxidase, hs-CRP and IL-10 were determined. In addition to decreased body weight and reduced glucose and HbA1c levels, we observed a reduction in superoxide production in leukocytes of diabetic patients and increased glutathione content, prominently after 24 weeks of empagliflozin treatment. Leukocyte expression of glutathione s-reductase and catalase, and serum levels of IL-10 were enhanced at 24 weeks of empagliflozin treatment. Concomitantly, reduced hs-CRP and myeloperoxidase levels were seen. This study provides evidence of the antioxidant and anti-inflammatory properties of empagliflozin treatment in humans, which may contribute to its beneficial cardiovascular effects.

## 1. Introduction

Type 2 diabetes (T2D) is one of the most common chronic metabolic diseases, strongly associated with cardiovascular diseases, which account for up to 80% of deaths among the diabetic population, thus constituting a serious health problem [1,2,3]. The characteristic metabolic disturbances of T2D are insulin resistance, hyperglycaemia and altered lipid metabolism, which sustain a state of subacute chronic inflammation [4]. At the cellular level, these alterations lead to a shift in the balance between oxidants and antioxidants in favour of oxidants, such as superoxide, which is released mostly as a by-product of mitochondrial oxidative phosphorylation [5]. Antioxidant defenses include many enzymes, such as glutathione s-reductase (GSR), involved in the reduction of glutathione disulphide to glutathione (GSH), or catalase (CAT), which dismutates H_2_O_2_ into H_2_O and O_2_.

On the other hand, the systemic inflammation present in diabetic patients is mainly manifested by increased circulating pro-inflammatory cytokines, such as tumor necrosis factor alpha (TNFα) or interleukin 1 beta (IL-1β), released by leukocytes [6]. These two main features of diabetes, namely, oxidative stress and chronic inflammation are of pivotal importance for diabetes-induced cardiovascular risk, and as such are key targets for therapy [7].

A key player in the multifactorial pathophysiology of diabetes is glucose reabsorption by the kidneys, which becomes maladaptive and markedly enhanced, thereby contributing to hyperglycaemia [8]. The sodium and glucose co-transporter 2 (SGLT2), located at the kidney proximal tubule, is the main driver of glucose transport from urine back into the bloodstream, accounting for at least ninety percent of filtered glucose [9]. Recently, major attention has been focused on a novel type of oral anti-diabetic drugs (OAD), the SGLT2 inhibitors (iSGLT2). iSGLT2 act directly on glucose without depending on β-cell function or insulin resistance [10]. Empagliflozin is one such iSGLT2, and is being widely used both as monotherapy or combined with insulin or other OADs, as its mechanism of action does not interfere with traditional antidiabetic medications [11]. In addition to their antihyperglycaemic effects, iSGLT2s have been shown to improve tissue insulin sensitivity [12,13], increase glucagon secretion by alpha cells in the pancreas [14], prevent obesity by reducing body weight [15], and decrease blood pressure [16]. Most importantly, a reduction in cardiovascular mortality attributed to iSGLT2 treatment has been demonstrated in diabetic patients, in which a combined effect of the aforementioned mechanisms may be critical [17,18]. However, the precise molecular basis for cardiovascular protection in patients treated with iSGLT2 eludes our understanding.

Given this scenario, the aim of the present study was to examine the metabolic outcomes of empagliflozin treatment in T2D patients, focusing on its chronic (24 weeks of treatment) effects on inflammatory and oxidative stress signaling.

## 2. Experimental Section

### 2.1. Patients and Sample Collection

This is a post-authorization, observational, prospective follow-up study of a cohort of patients with T2D. The T2D diagnosis was confirmed according to the American Diabetes Association’s criteria [19].

Fifteen patients with T2D who received treatment with empagliflozin according to standard clinical care for T2D and who met the inclusion and exclusion criteria of the study were recruited from the Endocrinology Service (University Hospital Doctor Peset). Initially, 17 patients were included in the study, however, two were excluded due to lack of adherence to the treatment.

The inclusion criteria were as follows: age between 40 and 70 years of age and time of evolution of the disease greater than 10 years. Exclusion criteria were the following: significant renal impairment (creatinine >1.5 mg/dL or eGFR < 60 mL/min/1.73 m^2^); severe diabetic neuropathy; chronic diseases other than those directly related to cardiovascular risk; morbid obesity (BMI > 40 kg/m^2^); smoking habit or habitual alcohol intake. Patients were also excluded if other hypoglycaemic, antihypertensive or lipid-lowering treatment had been altered (changes in dose or type of drug) in the previous year or during the study period. The treatment consisted of 10 mg/day of empagliflozin for 24 weeks taken orally, and measurements were taken at baseline and at 12 weeks and 24 weeks. Given the safety and tolerability of empagliflozin [20] in patients with type 2 diabetes, and the efficacy demonstrated in large-scale clinical trials [17,18], the dose of 10 mg/day was chosen. Urinary tract infections and genital infections are the most common side effects for iSGLT2 treatment, and none of the subjects developed any of these conditions in the study period.

The study was performed in compliance with the statement of ethical principles for medical research of the Declaration of Helsinki and approval was obtained from our hospital’s Ethics Committee (CEIC 30/17) for all procedures involving human subjects. Informed written consents were obtained from all patients before their participation.

### 2.2. Anthropometric and Biochemical Analyses

During each visit patients underwent a physical examination to obtain anthropometrical measurements such as weight (kg), height (m), waist circumference (cm), systolic (SBP) and diastolic blood pressure (DBP, mmHg). Overnight fasting blood was collected from the antecubital vein between 8:00 a.m. and 9:30 a.m. Samples were centrifuged at 1500 g for 10 min at 4 °C to obtain serum for biochemical and molecular determinations.

Glucose levels were measured using enzymatic techniques and a Dax-72 autoanalyzer (Bayer Diagnostic, Tarrytown, NY, USA). Fasting insulin levels were quantified by immunochemiluminescence (Abbott, Madrid, Spain). Homeostasis model assessment of insulin resistance index (HOMA-IR) was estimated as (fasting insulin (μU/mL) × fasting glucose (mg/dL))/405. The percentage of HbA1c was obtained with an automatic glycohaemoglobin analyzer (Arkray Inc., Kioto, Japan).

The lipid profile was analysed as follows: total cholesterol and triglyceride levels were obtained by means of enzymatic assay, high-density lipoprotein concentration (HDL-c) was determined with a Beckman LX20 analyzer (Beckman Corp., Brea, CA, USA), and low-density lipoprotein concentration (LDL-c) was calculated with Friedewald’s formula. High sensitive C-reactive protein (hs-CRP) concentration was assessed by latex-enhanced immunonephelometric assay (Behring Nephelometer II—Dade Bhering, Inc., Newark, DE, USA).

### 2.3. Leukocyte Extraction

Blood collected in heparinized tubes was incubated for 45 min with dextran (3% *w*/*v* in saline solution; Sigma Aldrich, St. Louis, MO, USA). A gradient centrifugation was performed by placing collected supernatants on Ficoll-Hypaque (GE Healthcare, Chicago, IL, USA) and centrifuging them at 650 g for 25 min at room temperature. A bottom phase containing leukocytes was collected and centrifuged at 650 g for 10 min at room temperature. The pellets were collected, washed with phosphate buffered saline (PBS; Sigma Aldrich, St. Louis, MO, USA) and resuspended in Hank’s Balanced Salt Solution (HBSS; Sigma Aldrich, St. Louis, MO, USA). Pellets were counted and divided into aliquots and used fresh or stored at −80 °C until utilization.

### 2.4. Superoxide Production and GSH Content

MitoSOX Red mitochondrial superoxide indicator—MitoSOX™—(MTX, 5 × 10^−6^ mol/L; Thermo Fisher Scientific, Waltham, (Massachusetts), USA) was employed to evaluate mitochondrial superoxide oxidation, and CellTracker™ Green 5-chloromethylfluorescein diacetate (CMFDA, 1 × 10^−6^ mol/L; Thermo Fisher Scientific, Waltham, (Massachusetts), USA) was used to assess GSH content. After extraction, 1.5 × 10^5^ of fresh isolated leukocytes were seeded per well in 48-well plates and incubated with the corresponding fluorescent probes for 30 min. Cells from each subject were seeded in triplicate and 12 images per well were recorded. Fluorescence was analyzed with a fluorescence microscope (IX81; Olympus Corporation, Tokyo, Japan) coupled with the static cytometry software “ScanR” (Olympus). In order to visualize nuclei, we coupled both fluorochromes with Hoechst 33342 (4 × 10^−6^ mol/L, Sigma Aldrich, St. Louis (Missouri), USA).

Fluorescence was standardized and referred to as percentage of control.

### 2.5. Quantitative Analysis of GSR and CAT Gene Expression

Total RNA was extracted from the pellet of leukocytes using GeneAll Ribospin™ Total RNA extraction kit (Geneall Biotechnology, Hilden, Germany) following the manufacturer’s protocol.

Quantitative RT-PCR (qRT-PCR) analysis was carried out using FastStart Universal SYBR Green Master (Sigma Aldrich, St. Louis (Missouri), USA) and the 7500 Fast RT-PCR system (Life Technologies, Carlsbad, CA, USA). The purity and concentration of the RNA in samples was ascertained and calculated using a NanoDrop 200c spectrophotometer (Life Technologies, Thermo Fisher Scientific) at an absorbance of 260 nm/280 nm (A260/280).

First strand cDNA was attained by means of a RevertAid first-strand cDNA synthesis kit (Life Technologies, Thermo Fisher Scientific). qRT-PCR reactions were carried out as follows: 10 min at 95 °C, 40 cycles (designed in one step) at 95 °C for 10 s and one cycle at 60 °C for 30 s, and a melting curve stage.

Primers used for the study of mRNA expression of GSR were: Forward, GTGGAGGTGCTGAAGTTCTCC and Reverse, AACCATGCTGACTTCCAAGC; and for CAT were: Forward, CTTCGACCCAAGCAACATGC and Reverse, CGGTGAGTGTCAGGATAGGC. Data were normalized to GAPDH by using the primers: Forward, CGCATCTTCTTTTGCGTCG and Reverse, TTGAGGTCAATGAAGGGGTCA. Relative quantification was carried out according to the comparative 2^−ΔΔCt^ method. Data were analyzed with Expression Suite software (Life Technologies, Thermo Fisher Scientific).

### 2.6. Myeloperoxidase (MPO) and Interleukin 10 (IL-10) Levels

MPO and IL-10 levels were evaluated in serum using a Luminex 200 flow analyzer system (Millipore, Austin, TX, USA).

### 2.7. Statistical Analysis

GraphPad Prism version 7.00 (GraphPad Software, La Jolla, CA, USA, www.graphpad.com) was employed for statistical analysis. Parametric data are expressed as mean ± standard deviation (SD) and non-parametric variables as median (25th and 75th percentiles). Bar graphs show mean ± standard error of the mean (SEM). The three groups were subjected to repeated measure one-way ANOVA analysis followed by Tukey’s multiple comparison test or the Friedman test followed by Dunn’s multiple comparisons test for parametric and non-parametric data. When only 2 groups were compared, a paired t-test was employed.

Differences were considered significant when *p* < 0.05.

## 3. Results

### 3.1. Anthropometric and Biochemical Parameters

The present study involved 15 diabetic subjects who initiated treatment with empagliflozin according to the standard clinical care for T2D. Among these patients, five were on treatment with metformin, one with insulin, four with metformin + DPP-4 inhibitors, two with metformin + DPP-4 inhibitors + insulin, two with metformin + insulin, and one patient received empagliflozin as monotreatment. However, all patients had received stable glucose-lowering therapy for at least 12 months before the study and continued to do so during follow-up; hence, empagliflozin treatment was the only substantial change in their treatment. With regard to lipid-lowering drugs, 80% of patients received statins. A total of nine patients took antihypertensive medications, five of which with diuretic treatment. Retinopathy and nephropathy affected 20% and 33% of T2D patients, respectively.

Anthropometric and metabolic characteristics of the study group are shown in Table 1. We observed a significant decrease in weight after 12 weeks of empagliflozin treatment (*p <* 0.05), and a further decrease at 24 weeks of treatment (*p <* 0.05 compared to baseline, and also between 12 and 24 weeks), together with a marked reduction in waist circumference (*p <* 0.01 between baseline and 24 weeks). Systolic and diastolic blood pressure remained unchanged during the follow-up period.

**Figure 1:**
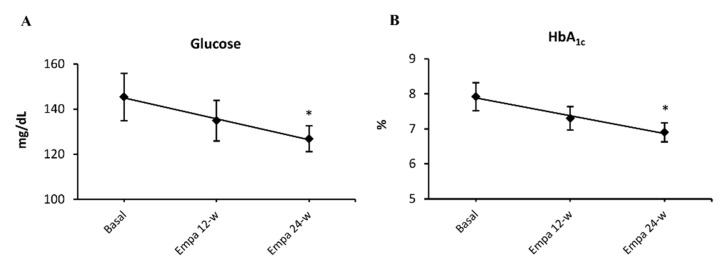
Empagliflozin effect on glucose metabolism. Blood glucose (**A**) and HbA1c levels (**B**) at 12 and at 24 weeks of treatment with empagliflozin 10 mg/day. Abbreviations: EMPA, empagliflozin; HbA1c %, glycated hemoglobin. * *p* < 0.05 vs. type 2 diabetes baseline.

Regarding lipid profile, our data showed a modest increase in total cholesterol at 12-weeks of empagliflozin treatment (*p <* 0.05), but this increase was not maintained at 24-weeks, though levels were still higher than at baseline. LDL-c and HDL-c levels increased slightly, but did not show significant differences after treatment. Triglyceride levels increased transiently at 12 weeks of treatment (*p <* 0.05). Lastly, when we analyzed insulin levels and HOMA-IR index, no differences from baseline were noted after treatment with empagliflozin. Patients whose treatment included insulin were excluded from HOMA-IR and insulin assessments. As expected, glucose levels decreased with empagliflozin treatment and became significant at 24-weeks of treatment (*p <* 0.05; Figure 1A). We observed the same pattern in HbA1c % (*p* = 0.09 at 12-weeks and *p <* 0.05 at 24-weeks treatment; Figure 1B).

### 3.2. Mitochondrial Superoxide Production and GSH Content in Leukocytes

**Figure 2:**
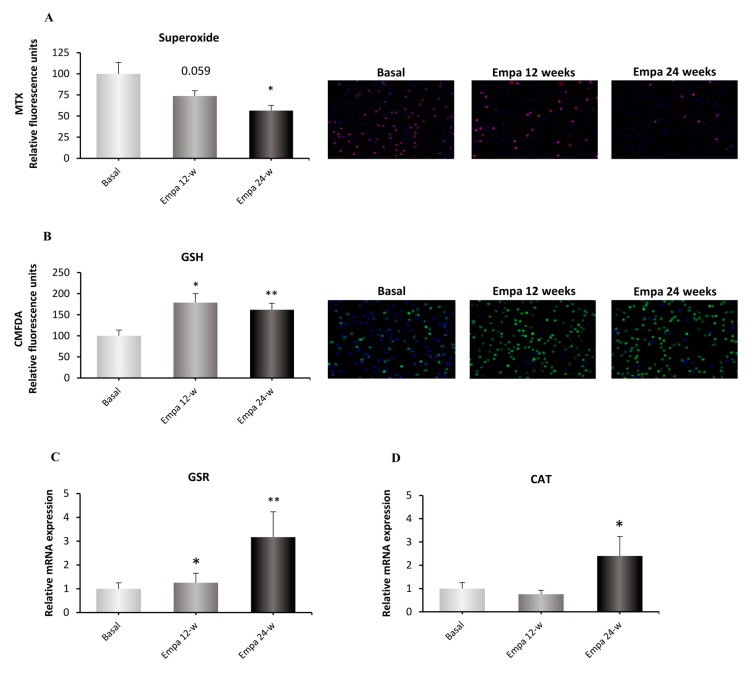
Effect of empagliflozin treatment on oxidative stress parameters and antioxidant enzymes. Mitochondrial superoxide production (**A**), GSH content (**B**) and mRNA expression of the antioxidant enzymes GSR (**C**) and CAT (**D**) in human leukocytes at 12 and 24 weeks of empagliflozin treatment. Data were normalized to fluorescence at baseline. The values of the relative gene expression were normalized to baseline mRNA expression levels and calculated using the 2^−ΔΔCT^ method. Abbreviations: EMPA, empagliflozin; MTX, MitoSOX Red mitochondrial superoxide indicator; CMFDA, 5-chloromethylfluorescein diacetate; GSH, glutathione, GSR, glutathione S-reductase; CAT, catalase. * *p* < 0.05, ** *p* < 0.05 vs. type 2 diabetes baseline. Representative fluorescence microscopy images are shown.

Data showed a tendency (*p* = 0.059) to produce less mitochondrial superoxide after 12 weeks, a value that became statistically significant after 24 weeks (*p <* 0.05; Figure 2A) and is indicative of a reduction in oxidative stress.

GSH content, an endogenous antioxidant, increased significantly after 12 weeks of treatment (*p <* 0.05) and this trend was maintained after 24 weeks (*p <* 0.01; Figure 2B). Despite this antioxidant marker improvement, plasma carbonyl content did not change after either 12 or 24 weeks of empagliflozin treatment (data not shown).

### 3.3. Antioxidant Gene Expression

Besides GSH content, we evaluated GSR gene expression, which codifies for the enzyme involved in GSH synthesis. As shown in Figure 2C, GSR levels increased significantly after 24 weeks of treatment (*p* < 0.05) compared to baseline. The same pattern was observed for the mRNA expression of CAT, another key antioxidant enzyme, which augmented in a significant manner after 24 weeks of treatment (*p <* 0.05; Figure 2D).

### 3.4. Inflammatory Parameters

**Figure 3:**
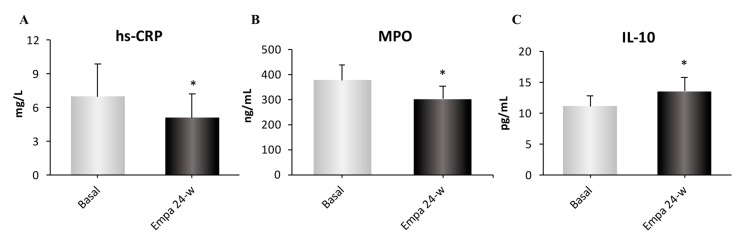
Assessment of inflammatory parameters; serum hs-CRP (**A**), MPO (**B**) and IL-10 (**C**) levels at baseline, 12 and 24 weeks of treatment. Abbreviations: EMPA, empagliflozin; hs-CRP, high-sensitive C-reactive protein; MPO, myeloperoxidase; IL-10, interleukin 10. * *p* < 0.05 compared with type 2 diabetes baseline.

Because the main effects of empagliflozin on metabolism and oxidative stress were evident at 24 weeks of treatment, we focused on inflammatory markers at this time point. Analyzing the pro-inflammatory markers hs-CRP and MPO, we observed a significant decrease in both markers after treatment (both *p <* 0.05; Figure 3A,B). In terms of the anti-inflammatory cytokine IL-10, we observed a significant increase in its levels following treatment (*p <* 0.05; Figure 3C).

## 4. Discussion

iSGLT2s are a new class of oral hypoglycaemic drugs that block glucose reabsorption at the renal level, thus promoting urine glucose and sodium excretion and reducing plasma glucose levels [21]. In the present study, we have focused on the effects of empagliflozin on oxidative stress in the leukocytes of T2D subjects, and on inflammatory parameters. In addition to a reduction in body weight and an improved metabolic profile—characterized by reduced plasma glucose and HbA1c levels—we observed a reduction in mitochondrial superoxide production in diabetic patients after treatment with the iSGLT2 empagliflozin, together with increased antioxidant defenses. Moreover, reduced proinflammatory markers and increased anti-inflammatory parameters were maintained after 24 weeks of treatment with empagliflozin. Besides their glucose-lowering effect, iSGLT2s produce changes in the lipid profile that should also be highlighted. Empagliflozin treatment is correlated with increased total cholesterol due to slight increases in HDL-c and LDL-c, as we have observed, and reported in other studies [22,23]. However, there is a well-documented cardiovascular protection exerted by iSGLT2s, which endows these drugs with clinical potential [24]. This has been reflected by several studies, such as the EMPA-REG OUTCOME study [25] in which a reduction of 38% in cardiovascular mortality was evident in the empagliflozin vs. placebo group. However, neither the reduction in HbA1c observed in the study (0.60% over 12 weeks) nor the reduction in SBP (up to 5 mmHg) are sufficient to explain the reported protection against cardiovascular mortality [26,27].

Interestingly, as little as 24 weeks of treatment with iSGLT2 is sufficient to witness their beneficial effects on HbA1c, body weight, blood pressure and, most importantly, on the cardiovascular system [28]. As an example, the reduction in hospitalization rates for heart failure in the group treated with empagliflozin in the EMPA-REG OUTCOME study was already significant at this time point [25]. A recent study demonstrated an improved arterial stiffness after 6 weeks of treatment with empagliflozin in T2D patients, and hs-CRP was shown to be one of the main significant determinants for this improvement [29]. Nevertheless, despite the available evidence, the molecular mechanisms underlying the cardiovascular protection attributable to iSGLT2 treatment are still unknown, though amelioration of oxidative stress has been hypothesized to be a potential contributor [30]. Because cardiac tissue possesses low antioxidant defenses, increasing the expression or activity of antioxidant enzymes could be a mechanism of cardiac protection under high risk situations such as T2D.

Oxidative stress occurs when the production of pro-oxidant species overcomes the intrinsic antioxidant defense system of the cell, thus triggering damage to lipids, proteins and DNA, and eventually compromising cellular and tissue function. Exploring the role of SGLT2 inhibition in the prevention or reduction of oxidative stress conditions has been the subject of several studies over the last decade. However, to date, most of the studies evaluating the effect of iSGLT2 on oxidative stress have been performed in animal models [31,32,33].

Our data demonstrate that T2D patients receiving iSGLT2 treatment undergo a reduction in mitochondrial superoxide production in parallel to an increase in glutathione content, and markedly so after 24 weeks of treatment. This is accompanied by increased expression of the antioxidant enzymes GSR and CAT. However, no changes in plasma protein carbonyl content were observed with empagliflozin treatment at these time-points. Whether this could be due to the small sample size or would require long-term empagliflozin treatment needs to be addressed in future research. Our findings are in accordance with previous studies in rat models of T2D, in which increased antioxidant enzyme content [32] and activity [33] in renal tissues has been reported. Furthermore, iSGLT2 treatment in diabetic mice has been shown to induce antioxidant gene expression (manganese-dependent superoxide dismutase and CAT) in adipose tissue and muscle [34].

Our study provides consistent and novel evidence of the antioxidant effect of empagliflozin treatment in humans. In this regard, one study showed that dapagliflozin, another iSGLT2, promoted a reduction in urinary isoprostane excretion, an indirect marker of oxidative stress, although this effect was acute (2 days of dapagliflozin treatment) and chronic effects were not evaluated [35]. Regarding empagliflozin, a previous study in patients with T2D showed that the use of empagliflozin was associated with a reduction in oxidative stress, although the study’s duration was rather short (28 days). Moreover, changes were evident in a single marker of oxidative stress—namely, the urinary excretion of 8-iso-prostaglandin F2α—but neither reactive oxygen species production nor antioxidant content were studied in cells isolated from such patients [36]. We have focused our study on immune cells, which are key players in the context of metabolic diseases, and particularly in T2D [6]. Circulating leukocytes participate in the maintenance of a chronic low-grade inflammatory state that is ongoing during T2D and could be one of the main causes of increased cardiovascular risk in these patients. Moreover, T2D patients are characterized by increased circulating proinflammatory cytokines and decreased anti-inflammatory IL-10, with these molecules being released by leukocytes [37,38,39]. We observed decreased inflammation following 24 weeks of empagliflozin treatment, evidenced by a drop in circulating hs-PCR. In addition, serum myeloperoxidase concentration was also markedly reduced by iSGLT2. This pro-oxidant enzyme is mainly released by neutrophils as an anti-microbial response, but enhanced levels have been observed during chronic inflammatory situations, and may trigger damage to the vascular walls [40,41]. We have previously demonstrated higher levels of myeloperoxidase in T2D patients, especially in those with vascular complications [42].

The present study, indicates that myeloperoxidase levels are reduced with empagliflozin, suggesting that this may be the way in which empagliflozin prevents the oxidative damage to the endothelium that precedes many of the complications of diabetes. We observed a concomitant increase in the anti-inflammatory cytokine IL-10 after 24 weeks of empagliflozin treatment. This finding supports an anti-inflammatory role of iSGLT2, which could contribute to preserving the vascular system in T2D patients. Recently, an ex vivo study demonstrated that SGLT2 inhibition prevents the hyperglycaemia-induced impairment of endothelium-dependent vasodilatation [43]. In accordance with these potential benefits to the vascular system, animal studies have shown that empagliflozin protects against vascular complications by undermining oxidative stress [31,44], which is reinforced by our present findings.

Is important to take into account that empagliflozin may decrease hs-CRP and reduce the levels of lipoproteins via ameliorating insulin resistance [45]. Therefore, the cardiovascular benefits conferred by empagliflozin might be driven at least partly by anti-inflammatory effects, and this mechanism might cooperate with reduced blood pressure, also observed under empagliflozin treatment [45]. The underlying mechanisms to explain empagliflozin-induced anti-inflammatory effects are multiple, but may involve weight loss, a reduction in adipose tissue inflammation, a slight increase in ketone bodies, and diminution of uric acid levels or attenuation of oxidative stress [46].

## 5. Conclusions

In summary, this study provides new evidence of the antioxidant and anti-inflammatory properties of empagliflozin treatment in humans. These benefits may play a role in the well documented cardiovascular protection provided by iSGLT2, although the precise underlying mechanisms require further investigation.

## Figures and Tables

**Table 1:** Anthropometric parameters of the study population at baseline, 12 weeks and at the end of 24-week follow-up.

	Baseline	12 Weeks Empagliflozin	24 Weeks Empagliflozin	*p*-Value
*N* (males)	15 (11)	15 (11)	15 (11)	
Age (years)	60.8 ± 10.2	-	-	
Weight (kg)	88.1 ± 19.8	85.1 ± 20.2 *	83.7 ± 20.4 *^#^	<0.001
Waist circumference (cm)	104.4 ± 12.4	100.9 ± 15.6	98.22 ± 13.0 **	<0.05
SBP (mmHg)	139.3 ± 25.9	137.3 ± 20.9	139.3 ± 21.2	ns
DBP (mmHg)	77.2 ± 11.6	79.1 ± 13.3	77.0 ± 12.5	ns
Total cholesterol (mg/dL)	141 ± 25	155 ± 28 *	150 ± 27	<0.05
LDL-c (mg/dL)	79.5 ± 18.6	89.2 ± 17.0	86.7 ± 20.4	ns
HDL-c (mg/dL)	44.6 ± 7.8	42.8 ± 6.9	47.0 ± 3.9	ns
Triglycerides (mg/dL)	94 (86−137)	119 (101−171) *	108 (85−130)	<0.05
Insulin (μUI/mL)	9.6 ± 5.7	9.3 ± 5.8	9.2 ± 6.1	ns
HOMA-IR	3.28 ± 2.23	3.00 ± 1.63	2.83 ± 1.96	ns

^1^ Data are expressed as mean ± SD for parametric variables and as median (interquartile range) for non-parametric data. Repeated measure one-way ANOVA followed by Tukey’s multiple comparisons test or Friedman test followed by Dunn’s multiple comparisons test were performed when comparing parametric and non-parametric variables, respectively. * *p* < 0.05 vs. baseline, ** *p* < 0.01 vs. baseline, ^#^ *p* < 0.05 vs. 12 weeks-empagliflozin. Abbreviations: SBP, systolic blood pressure; DBP, diastolic blood pressure; LDL-c, low-density lipoprotein cholesterol; HDL-c, high-density lipoprotein cholesterol; HOMA-IR, homeostasis model assessment of insulin resistance.

## References

[1] Lahnwong S., Chattipakorn S.C., Chattipakorn N. (2018). Potential mechanisms responsible for cardioprotective effects of sodium-glucose co-transporter 2 inhibitors. Curr. Diabetol..

[2] Gaede P., Lund-Andersen H., Parving H.H., Pedersen O. (2008). Effect of a multifactorial intervention on mortality in type 2 diabetes. N. Engl. J. Med..

[3] Mozaffarian D., Benjamin E.J., Go A.S., Arnett D.K., Blaha M.J., Cushman M., Das S.R., de Ferranti S., Després J.P., Fullerton H.J. (2016). Executive summary: Heart disease and stroke statistics—2016 update: A report from the American heart association. Circulation.

[4] Saltiel A.R., Olefsky J.M. (2017). Inflammatory mechanisms linking obesity and metabolic disease. J. Clin. Investig..

[5] Hernandez-Mijares A., Rocha M., Rovira-Llopis S., Bañuls C., Bellod L., De Pablo C., Alvarez A., Roldan-Torres I., Sola-Izquierdo E., Victor V.M. (2013). Human leukocyte/endothelial cell interactions and mitochondrial dysfunction in type 2 diabetic patients and their association with silent myocardial ischemia. Diabetes Care.

[6] Lackey D.E., Olefsky J.M. (2016). Regulation of metabolism by the innate immune system. Nat. Rev. Endocrinol..

[7] Burgos-Morón E., Abad-Jiménez Z., Marañón A.M., Iannantuoni F., Escribano-López I., López-Domènech S., Salom C., Jover A., Mora V., Roldan I. (2019). Relationship Between Oxidative Stress, ER Stress, and Inflammation in Type 2 Diabetes: The Battle Continues. J. Clin. Med..

[8] DeFronzo R.A. (2009). From the triumvirate to the ominous octet: A new paradigm for the treatment of type 2 diabetes mellitus. Diabetes.

[9] Anders H.J., Davis J.M., Thurau K. (2016). Nephron protection in diabetic kidney disease. N. Engl. J. Med..

[10] Wright E.M. (2001). Renal Na(+)-glucose cotransporters. Am. J. Physiol. Renal Physiol..

[11] Abdul-Ghani M.A., Norton L., Defronzo R.A. (2011). Role of sodium-glucose cotransporter 2 (SGLT 2) inhibitors in the treatment of type 2 diabetes. Endocr. Rev..

[12] Ferrannini E., Muscelli E., Frascerra S., Baldi S., Mari A., Heise T., Broedl U.C., Woerle H.J. (2014). Metabolic response to sodium glucose cotransporter 2 inhibition in type 2 diabetic patients. J. Clin. Investig..

[13] Merovci A., Solis-Herrera C., Daniele G., Eldor R., Fiorentino T.V., Tripathy D., Xiong J., Perez Z., Norton L., Abdul-Ghani M.A. (2014). Dapagliflozin improves muscle insulin sensitivity but enhances endogenous glucose production. J. Clin. Investig..

[14] Bonner C., Kerr-Conte J., Gmyr V., Gmyr V., Queniat G., Moerman E., Thévenet J., Beaucamps C., Delalleau N., Popescu I. (2015). Inhibition of the glucose transporter SGLT2 with dapagliflozin in pancreatic alpha cells triggers glucagon secretion. Nat. Med..

[15] Ferrannini G., Hach T., Crowe S., Sanghvi A., Hall K.D., Ferrannini E. (2015). Energy balance after sodium-glucose cotransporter 2 inhibition. Diabetes Care.

[16] Rajasekeran H., Lytvyn Y., Cherney D.Z.I. (2016). Sodium–glucose cotransporter 2 inhibition and cardiovascular risk reduction in patients with type 2 diabetes: The emerging role of natriuresis. Kidney Int..

[17] Wanner C., Inzucchi S.E., Zinman B. (2016). Empagliflozin and progression of kidney disease in Type 2 Diabetes. N. Engl. J. Med..

[18] Fitchett D., Inzucchi S.E., Cannon C.P., McGuire D.K., Scirica B.M., Johansen O.E., Sambevski S., Kaspers S., Pfarr E., George J.T. (2019). Empagliflozin reduced mortality and hospitalization for heart failure across the spectrum of cardiovascular risk in the EMPA-REG OUTCOME trial. Circulation.

[19] American Diabetes Association (2019). Classification and diagnosis of diabetes: Standards of medical care in diabetes-2019. Diabetes Care.

[20] Kohler S., Zeller C., Iliev H., Kaspers S. (2017). Safety and Tolerability of Empagliflozin in Patients with Type 2 Diabetes: Pooled Analysis of Phase I-III Clinical Trials. Adv. Ther..

[21] Sattar N., Petrie M.C., Zinman B., Januzzi J.L. (2017). Novel diabetes drugs and the cardiovascular specialist. J. Am. Coll. Cardiol..

[22] Monami M., Dicembrini I., Kundisova L., Zannoni S., Nreu B., Mannucci E. (2014). A meta-analysis of the hypoglycaemic risk in randomized controlled trials with sulphonylureas in patients with type 2 diabetes. Diabetes Obes. Metab..

[23] Häring H.U., Merker L., Seewaldt-Becker E., Weime M., Meinicke T., Broedl U.C., Woerle H.J. (2014). EMPA-REG MET Trial Investigators. Empagliflozin as add-on to metformin in patients with type 2 diabetes: A 24-week, randomized, double-blind, placebo-controlled trial. Diabetes Care.

[24] Zelniker T.A., Wiviott S.D., Raz I., Im K., Goodrich E.L., Bonaca M.P., Mosenzon O., Kato E.T., Cahn A., Furtado R.H.M. (2019). SGLT2 inhibitors for primary and secondary prevention of cardiovascular and renal outcomes in type 2 diabetes: A systematic review and meta-analysis of cardiovascular outcome trials. Lancet.

[25] Zinman B., Wanner C., Lachin J.M., Fitchett D., Bluhmki E., Hantel S., Mattheus M., Devins T., Johansen O.E., Woerle H.J. (2015). Empagliflozin, Cardiovascular outcomes, and mortality in type 2 diabetes. N. Engl. J. Med..

[26] Turnbull F.M., Abraira C., Anderson R.J., Byington R.P., Chalmers J.P., Duckworth W.C., Evans G.W., Gerstein H.C., Holman R.R., Moritz T.E. (2009). Intensive glucose control and macrovascular outcomes in type 2 diabetes. Diabetologia.

[27] Emdin C.A., Rahimi K., Neal B., Callender T., Perkovic V., Patel A. (2015). Blood pressure lowering in type 2 diabetes: A systematic review and meta-analysis. JAMA.

[28] Dalama B., Mesa J. (2016). New oral hypoglycemic agents and cardiovascular risk. Crossing the metabolic border. Rev. Esp. Cardiol..

[29] Bosch A., Ott C., Jung S., Striepe K., Karg M.V., Kannenkeril D., Dienemann T., Schmieder R.E. (2019). How does empagliflozin improve arterial stiffness in patients with type 2 diabetes mellitus? Sub analysis of a clinical trial. Cardiovasc. Diabetol..

[30] Yaribeygi H., Atkin S.L., Butler A.E., Sahebkar A. (2019). Sodium-glucose cotransporter inhibitors and oxidative stress: An update. J. Cell Physiol..

[31] Oelze M., Kröller-Schön S., Welschof P., Jansen T., Hausding M., Mikhed Y., Stamm P., Mader M., Zinßius E., Agdauletova S. (2014). The sodium-glucose co-transporter 2 inhibitor empagliflozin improves diabetes-induced vascular dysfunction in the streptozotocin diabetes rat model by interfering with oxidative stress and glucotoxicity. PLoS ONE.

[32] Shin S.J., Chung S., Kim S.J., Lee E.M., Yoo Y.H., Kim J.W., Ahn Y.B., Kim E.S., Moon S.D., Kim M.J. (2016). Effect of sodium-glucose co-transporter 2 inhibitor, dapagliflozin, on renal renin-angiotensin system in an animal model of type 2 diabetes. PLoS ONE.

[33] Osorio H., Coronel I., Arellano A., Pacheco U., Bautista R., Franco M., Escalante B. (2012). Sodium-glucose cotransporter inhibition prevents oxidative stress in the kidney of diabetic rats. Oxid. Med. Cell Longev..

[34] Sugizaki T., Zhu S., Guo G., Matsumoto A., Zhao J., Endo M., Horiguchi H., Morinaga J., Tian Z., Kadomatsu T. (2017). Treatment of diabetic mice with the SGLT2 inhibitor TA-1887 antagonizes diabetic cachexia and decreases mortality. NPJ Aging Mech. Dis..

[35] Solini A., Giannini L., Seghieri M., Vitolo E., Taddei S., Ghiadoni L., Bruno R.M. (2017). Dapagliflozin acutely improves endothelial dysfunction, reduces aortic stiffness and renal resistive index in type 2 diabetic patients: A pilot study. Cardiovasc. Diabetol..

[36] Nishimura R., Tanaka Y., Koiwai K., Inoue K., Hach T., Salsali A., Lund S.S., Broedl U.C. (2015). Effect of empagliflozin monotherapy on postprandial glucose and 24-h glucose variability in Japanese patients with type 2 diabetes mellitus: A randomized, double-blind, placebo-controlled, 4-week study. Cardiovasc. Diabetol..

[37] Rovira-Llopis S., Bañuls C., de Marañon A.M., Diaz-Morales N., Jover A., Garzon S., Rocha M., Victor V.M., Hernandez-Mijares A. (2017). Low testosterone levels are related to oxidative stress, mitochondrial dysfunction and altered subclinical atherosclerotic markers in type 2 diabetic male patients. Free Radic. Biol. Med..

[38] Van Exel E., Gussekloo J., de Craen A.J., Frölich M., Bootsma-Van Der Wiel A., Westendorp R.G. (2002). Leiden 85 Plus Study. Low production capacity of interleukin-10 associates with the metabolic syndrome and type 2 diabetes: The Leiden 85-Plus Study. Diabetes.

[39] Tedgui A., Mallat Z. (2006). Cytokines in atherosclerosis: Pathogenic and regulatory pathways. Physiol. Rev..

[40] Baldus S., Heeschen C., Meinertz T., Zeiher A.M., Eiserich J.P., Münzel T., Simoons M.L., Hamm C.W., CAPTURE Investigators (2003). Myeloperoxidase serum levels predict risk in patients with acute coronary syndromes. Circulation.

[41] Victor V.M., Rovira-Llopis S., Bañuls C., Diaz-Morales N., Martinez de Marañon A., Rios-Navarro C., Alvarez A., Gomez M., Rocha M., Hernández-Mijares A. (2016). Insulin resistance in PCOS patients enhances oxidative stress and leukocyte adhesion: Role of myeloperoxidase. PLoS ONE.

[42] Rovira-Llopis S., Rocha M., Falcon R., de Pablo C., Alvarez A., Jover A., Hernandez-Mijares A., Victor V.M. (2013). Is myeloperoxidase a key component in the ROS-induced vascular damage related to nephropathy in type 2 diabetes?. Antioxid Redox Signal..

[43] El-Daly M., Pulakazhi Venu V.K., Saifeddine M., Mihara K., Kang S., Fedak P.W.M., Alston L.A., Hirota S.A., Ding H., Triggle C.R. (2018). Hyperglycaemic impairment of PAR2-mediated vasodilation: Prevention by inhibition of aortic endothelial sodium-glucose-co-Transporter-2 and minimizing oxidative stress. Vascul. Pharmacol..

[44] Steven S., Oelze M., Hanf A., Kröller-Schön S., Kashani F., Roohani S., Welschof P., Kopp M., Gödtel-Armbrust U., Xia N. (2017). The SGLT2 inhibitor empagliflozin improves the primary diabetic complications in ZDF rats. Redox Biol..

[45] Hattori S. (2018). Anti-inflammatory effects of empagliflozin in patients with type 2 diabetes and insulin resistance. Diabetol. Metab. Syndr..

[46] Hadjadj S., Rosenstock J., Meinicke T., Woerle H.J., Broedl U.C. (2016). Initial Combination of Empagliflozin and Metformin in Patients with Type 2 Diabetes. Diabetes Care.

